# Thermal Analysis of the Receiver of a Standalone Pilot Solar Dish–Stirling System

**DOI:** 10.3390/e20060429

**Published:** 2018-06-04

**Authors:** Ehsan Gholamalizadeh, Jae Dong Chung

**Affiliations:** Department of Mechanical Engineering, Sejong University, Seoul 05006, Korea

**Keywords:** renewable energy, thermal analysis, solar thermal technology, solar dish-stirling system, receiver thermal efficiency

## Abstract

Recent developments in solar thermal systems have aroused considerable interest in several countries with high solar potential. One of the most promising solar driven technologies is the solar thermal dish-Stirling system. One of the main issues of the solar dish–Stirling system is thermal losses from its components. The majority of the thermal losses of the system occur through its receiver before the thermal energy is converted to electrical energy by the Stirling engine. The goal of this investigation is to analyze the thermal performance of the receiver of a standalone pilot solar dish–Stirling system installed in Kerman City, Iran, to be used in remote off-grid areas of the Kerman Province. An analytical model was developed to predict the input energy, thermal losses, and thermal efficiency of the receiver. The receiver thermal model was first validated by comparing simulation results to experimental measurements for the EuroDish project. Then, the incident flux intensity intercepted by the receiver aperture, the thermal losses through the receiver (including conduction, convection, and radiation losses), and the power output during daytime hours (average day of each month for a year) were predicted. The results showed that the conduction loss was small, while the convection and radiation losses played major roles in the total thermal losses through the receiver. The convection loss is dominant during the early morning and later evening hours, while radiation loss reaches its highest value near midday. Finally, the thermal efficiency of the receiver and the power output for each working hour throughout the year were calculated. The maximum performance of the system occurred at midday in the middle of July, with a predicted power output of 850 W, and a receiver efficiency of about 60%. At this time, a conduction loss of about 266 W, a convection loss of 284 W, and a radiation loss of about 2000 W were estimated.

## 1. Introduction

To date, several types of solar thermal technologies have been developed to produce electricity from solar energy. Currently, a parabolic solar dish–Stirling system is one of the types of concentrating solar power plants considered as one of the most efficient proven solar thermal technologies [1].

A parabolic solar dish–Stirling system has a solar collector that consists of a solar parabolic dish and a thermal receiver through which thermal energy is provided to drive a Stirling engine [2]. The parabolic dish concentrates solar radiation onto the aperture of the thermal receiver. The receiver includes an aperture and an absorber. The aperture is located at the focal point of the dish, at which the solar radiation reflected from the solar concentrator is the most concentrated. Then, the received thermal energy is transferred to the working fluid of a Stirling engine by the absorber. This input thermal energy is then converted into mechanical energy by the Stirling engine. Finally, a generator driven by the mechanical energy produces electricity.

**Figure 1:**
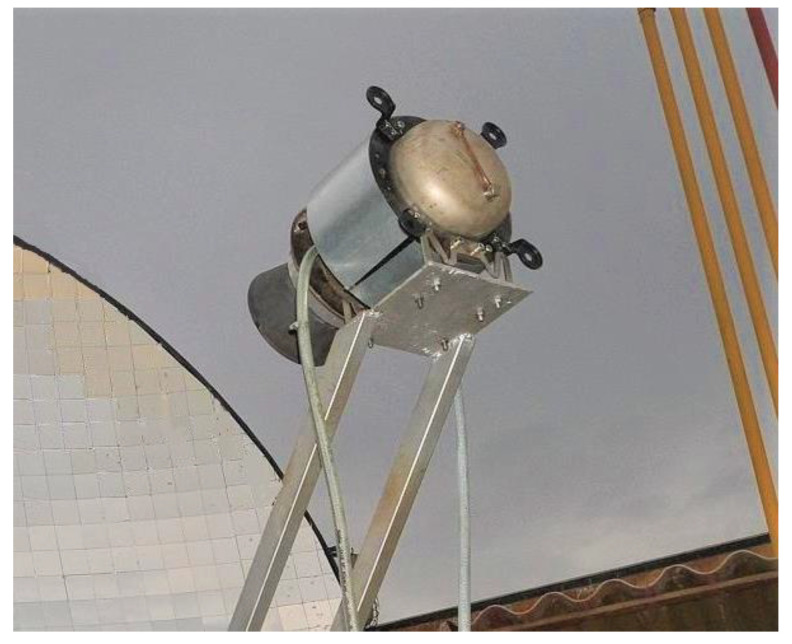
Kerman pilot dish–Stirling system.

This system is classified as the most efficient model, exceeding the total efficacy of any other solar conversion technology [1]; hence, countries with huge amounts of solar irradiation, such as the desert areas of Iran, are showing considerable interest. One such area is Kerman City (Iran; 30°17′ N, 57°5′ E), where the average solar irradiation is >2000 kWh/m^2^ year. The solar hours in this area are almost 2800 h/year [2]. A standalone pilot solar dish–Stirling system was set up under the meteorological conditions specific to Kerman City. The power produced was to be applied in remote areas where accessing the electrical grid is very difficult. The proposed module consists of a collector with a parabolic dish diameter of 3 m. It was manufactured using identical square 0.08 × 0.08 m glass/silver mirror panels 2-mm thick. The receiver aperture had a diameter of 0.12 m (i.e., C = 625), and a rim angle of 45°. A directly illuminated tube type was used for the receiver, so it can only be operated during the daytime (solar-only type). A free piston Stirling engine with a nominal capacity of 1 kW was installed in the Kerman pilot facility. The engine working fluid was helium, and the system was subjected to maximum temperature and pressure of 800 K and 10 bars, respectively. Figure 1 shows the receiver and the engine installed on the Kerman pilot.

So far, several investigations have been conducted to design and analyze a standalone dish Stirling module. One of the main issues being addressed by designers is the performance of the different parts of the system. Hence, a number of investigations have focused on modeling and improving solar dish–Stirling modules, in particular, the solar receiver.

The performance of a parabolic solar dish–Stirling system was examined by Wu et al. [3], who reported a power output and total efficiency of 18.54 kW and 20.6%, respectively. Nepveu et al. [4] developed a thermal model for the 10-kW EuroDish dish–Stirling unit. A mathematical model was developed to evaluate the overall thermal efficiency of the solar-powered high temperature differential dish–Stirling engine with finite-rate heat transfer, regenerative heat losses, conductive thermal bridging losses, and finite regeneration process time [5]. Producing 100 MW of electricity using solar dish–Stirling technology was examined [6] to evaluate the thermal energy and the levelized energy cost. The effect of the rim angle on the flux distribution diameter of the system was considered in Sup et al. [7]. A rim angle of 45° was proposed to obtain the highest thermal performance of the system collector [8]. In Gholamalizadeh and Chung [9], a pilot was installed which its collector was designed with a rim angle of 45°. Ruelas et al. [10] presented a new mathematical model and performed numerical examinations of a thermal model of a receptor, to evaluate the technical feasibility of attaching a Scheffler-type solar concentrator to a 3-kW Stirling engine. A solar thermal Stirling engine designed by Ahmadi [11] maximized the thermal efficiency and power output. A thermodynamic analysis of the network and stored heat in the regenerator of a Stirling engine was done using an isothermal model [12]. Li et al. proposed a receiver geometry that was intended to achieve higher thermal efficiency [13]. Hussain et al. [14] investigated various configurations of the cavity receiver to reduce thermal losses. Different cavity configurations were also studied, including: semi-spherical [15,16], cylindrical [17], and rectangular receivers [18]. Based on a literature review, the thermal performance of the receiver and concentrator was predicted by developing a mathematical model implemented in the programing software Matlab.

The present paper proposes an approach for analysis of the thermal efficiency, taking into account the thermal losses through the receiver of the standalone Kerman pilot dish–Stirling system under the specific meteorological conditions in Kerman City. The power is to be used in remote off-grid desert areas of Kerman Province. An analytical model was used to predict the conduction, convection, and radiation losses through the receiver. The calculations were performed for the daytime hours of an average day in each month of a year [19]. Then, using the results, the thermal efficiency of the receiver and the power output were calculated. 

The main objective of the study is to present a practical methodology for predicting the performance of each part of the solar dish–Stirling system, and then, to demonstrate that the methodology provides the necessary elements to design the system modules. This approach shows the impact and importance of each thermal mechanism on the efficiency of the receiver at different periods of time.

## 2. Methodology

### 2.1. The Collector Parameters

**Figure 2:**
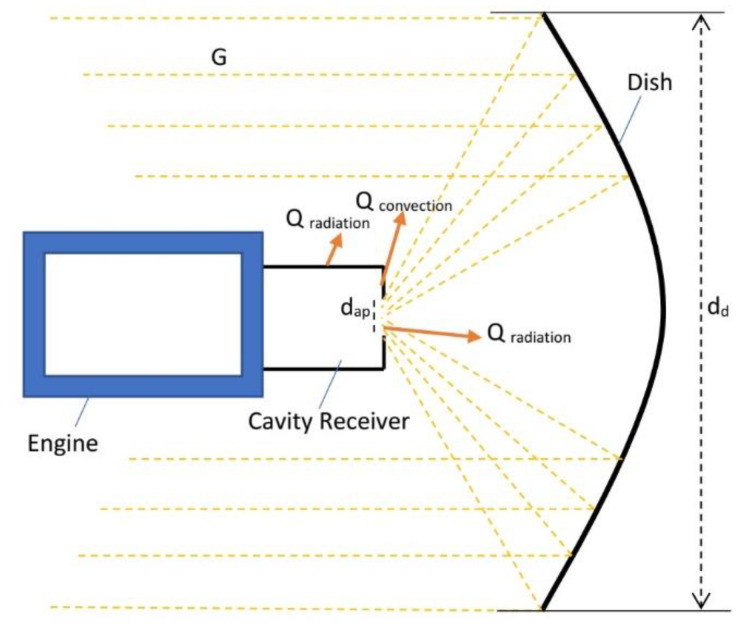
Schematic of the system.

The shape of a parabolic concentrator and the location of its focal point can be defined by the dish focal distance to diameter ratio (*f*/*d_d_*). This shape can also be defined by the rim angle. A schematic of the system is illustrated in Figure 2. 

The relationship between the focal distance and the rim angle is given below [20]:

(1) $$f=\frac{d_{d}}{4\tan(\psi_{rim}/2)}$$

The rim angle must be determined before sizing the aperture because it influences the maximum concentration ratio (CR), collector slope error, and losses due to convection and radiation [21]. 

The distance between the surface of the concentrator and the focal point of the aperture at any angle (*ψ*) between 0° and the rim angle is calculated as:

(2) $$Q=\frac{2f}{1+\cos(\psi )}$$

The CR can be defined as below:

(3) $$C\;={\left(\frac{d_{d}}{d_{ap}}\right)}^{2}$$

### 2.2. Thermal Modeling of the Receiver

A major fraction of the total system thermal losses occurs in the receiver and therefore, analyzing these losses is important. These losses include conduction through the receiver walls, radiation through the opening of the aperture to the environment, and convection from the cavity. 

#### 2.2.1. The Conduction Losses

The total conduction losses from the receiver can be estimated by [22]:

(4) $${\dot{Q}}_{conduction}=\frac{T_{cav}-T_{amb}}{ln\left[\left(\frac{d_{cav}}{2}+\delta_{insul}\right)/\frac{d_{cav}}{2}\right]/\left(2\pi \;k_{insul}L_{cav}\right)}$$

#### 2.2.2. The Convection Losses

The natural convective heat transfer coefficient, which refers to transfer through the receiver cavity, depends on the aperture and receiver diameters, and the cavity location on a specific day and at a specific time. To estimate this coefficient, the Nusselt number can be calculated as below [23]:

(5) $$Nu_{natural}=0.088\cdot Gr^{1/3}\cdot {\left(T_{cav}/T_{amb}\right)}^{0.18}\cdot {\left(\cos\theta \right)}^{2.47}\cdot {\left(d_{ap}/d_{cav}\right)}^{-0.982\;\cdot \;\left(d_{ap}/d_{cav}\right)\;+\;1.12}$$

The forced convective heat transfer coefficient of the receiver cavity can be expressed as a function of the wind speed, as follows [24]:

(6) $$h_{forced}=0.1967\cdot v^{1.849}$$

The total convective heat transfer coefficient and total convection losses through the receiver cavity are calculated using the following equations [24]:

(7) $$h_{total}=h_{natural}+h_{forced}$$

(8) $${\dot{Q}}_{convection}\;=h_{total}\cdot A_{cav}\cdot \left(T_{cav}-T_{amb}\right)$$

#### 2.2.3. The Radiation Losses

The radiation loss from the receiver is a considerable part of the total thermal losses of the receiver. It consists of two radiation mechanisms: the emitted radiation and the reflected radiation. The emitted and reflected radiation heat transfer from the receiver cavity is calculated with the following equations, respectively:

(9) $${\dot{Q}}_{emitted}=\varepsilon \cdot A_{ap}\cdot \sigma \cdot \left(T_{cav}^{4}-T_{amb}^{4}\right)$$

(10) $${\dot{Q}}_{reflected}=\left(1-\alpha_{eff}\right)\cdot \eta_{conc}\cdot G\cdot A_{d}$$

It is seen that the radiation loss reflected from the cavity surfaces depends on the effective absorptance of the cavity receiver, which is defined as $\alpha_{eff}=\alpha_{cav}/\left[\alpha_{cav}+\left(1-\alpha_{cav}\right)\left(A_{ap}/A_{cav}\right)\right]$. Therefore, the total radiation from the receiver cavity can be predicted by:

(11) $${\dot{Q}}_{radiation}={\dot{Q}}_{emitted}+{\dot{Q}}_{reflected}$$

#### 2.2.4. The Total Thermal Loss

The total thermal loss from the receiver is calculated as below:

(12) $${\dot{Q}}_{total,loss}={\dot{Q}}_{convection}+\;{\dot{Q}}_{conduction}+{\dot{Q}}_{radiation}$$

The receiver efficiency can be calculated as a function of the total thermal loss, the entrance solar energy, and the concentrator efficiency as follows:

(13) $$\eta_{\mathrm{rec}}=1-\frac{{\dot{Q}}_{total,loss}}{\eta_{conc}\cdot G\cdot A_{d}}$$

The heat rate transferred to the Stirling engine is assumed to be equal to the heat rate supplied by the receiver. Therefore, the thermal power input received by the Stirling engine can be estimated as below:

(14) $${\dot{Q}}_{SE}=\eta_{rec}\cdot \eta_{conc}\cdot G\cdot A_{d}$$

The total efficiency of the system is equal to the product of the concentrator efficiency (*η_conc_*), the receiver efficiency (*η_rec_*), the Stirling engine efficiency (*η_SE_*), and the generator efficiency (*η_gen_*) as below:

(15) $$\eta_{total}=\eta_{conc}\eta_{rec}\eta_{SE}\eta_{gen}$$

Finally, the power output of the system can be expressed as a product of the total input energy and the total efficiency as follows:

(16) $$P_{SE}=GA_{d}\;\eta_{total}$$

### 2.3. Solar Radiation Model

In order to calculate the annual energy that can be produced by the system, it is necessary to determine the amount of solar irradiance reaching the dish concentrator in a year. Since the aperture of the receiver is much smaller than the reflector, only beam radiation is effectively directed onto the absorbing surface of the receiver [25]; therefore, only beam radiation is taken into consideration in the simulations. The equations for estimating the beam solar irradiance intercepted by the concentrator are described in detail in Duffie and Beckman [25].

## 3. Results and Discussion

**Figure 3:**
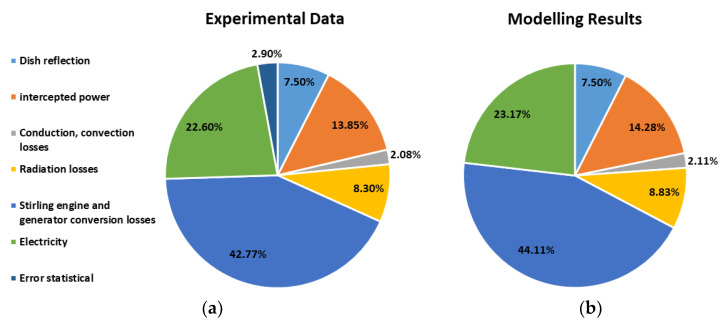
Comparison between measured data of the EuroDish project and simulation results.

The Kerman pilot produced about 0.6 kW of power output in the middle of June at the highest solar radiation, which was in good agreement with the predicted power output of 0.629 kW at the same time. In addition, the receiver thermal model simulation was validated by comparing its results to the measured data from the 10-kW EuroDish project [26]. Under the same operating conditions as the EuroDish project, the results obtained from the model are shown in Figure 3. This verified that the thermal model exhibited satisfactory agreement with the measured data.

**Figure 4:**
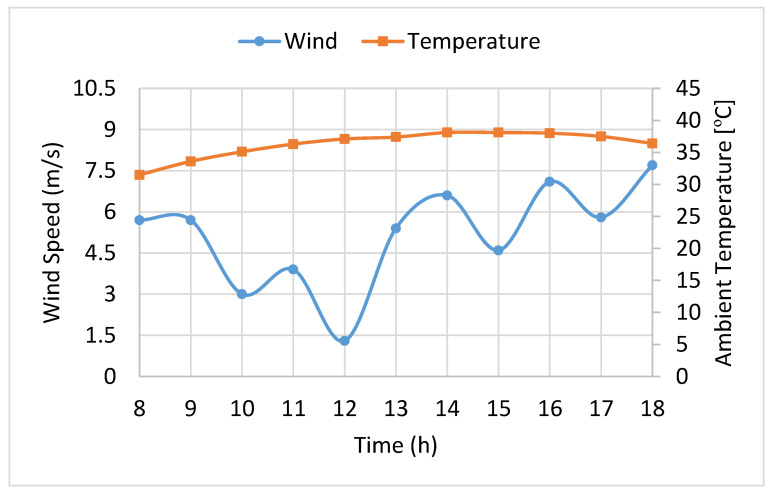
Wind speed and ambient temperature at the Kerman site recorded on 15 July.

The wind speed and the ambient temperature data recorded by the weather bureau of Kerman City throughout the year were used to take into account the effects of meteorological conditions of the site on the simulation results throughout the operating hours of the year. As an example, the data records of Kerman City during the working period of the pilot test on 15 July are reported in Figure 4.

Accordingly, the incident flux intensity intercepted by the receiver, the thermal losses, efficiency of the receiver, and system power output in the daytime hours of an average day for each month in a year were calculated. 

In this section, we report the results from our analysis of the thermal losses of the receiver, including the conduction, convection, and radiation losses. These losses are dependent on the location of the receiver, the receiver cavity temperature, and the time of day.

### 3.1. The Receiver Incident Flux Intensity

**Figure 5:**
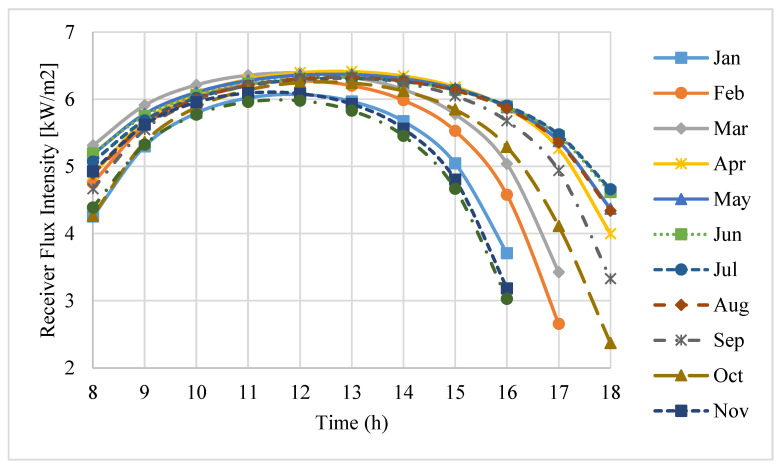
Receiver aperture incident flux intensity during the daytime hours on an average day of each month.

The incident flux intensity values intercepted by the receiver aperture in the daytime hours of an average day for each month in a year are shown in Figure 5. It can be seen that, from November to February, the flux intensity received by the receiver aperture is comparatively low. Moreover, during this period of time, the number of sunny hours in a day is also low. 

### 3.2. Conduction Loss through the Receiver

In order to minimize the conduction losses from the receiver cavity and also minimize the shading of the dish, an optimum insulation layer thickness of 75 mm for the high-temperature ceramic fiber insulation was suggested [22] and used in the receiver.

**Figure 6:**
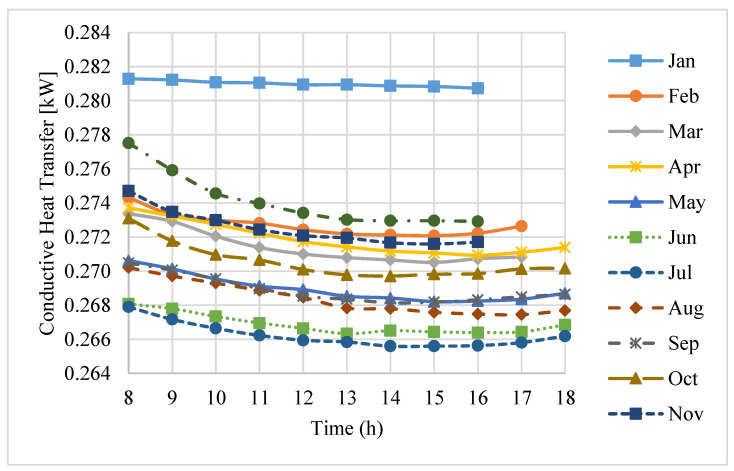
Conduction losses during the daytime hours on an average day of each month.

The total conduction loss in the working hours of an average day of each month in a year is shown in Figure 6. It can be seen that the losses change marginally with any change of the environmental conditions during the daytime. The model estimates an average total conduction loss of about 266 W to 281 W, which is a small portion of the total thermal losses. Moreover, it was found that the maximum thermal loss due to conduction occurs in January. In this month, the average conduction loss is about 5.3% of the average total losses. The main reason is that in this month, the ambient temperature at the site is much lower than in other months, which results in an increase in the convective heat transfer through the receiver.

### 3.3. Convection Loss through the Receiver

**Figure 7:**
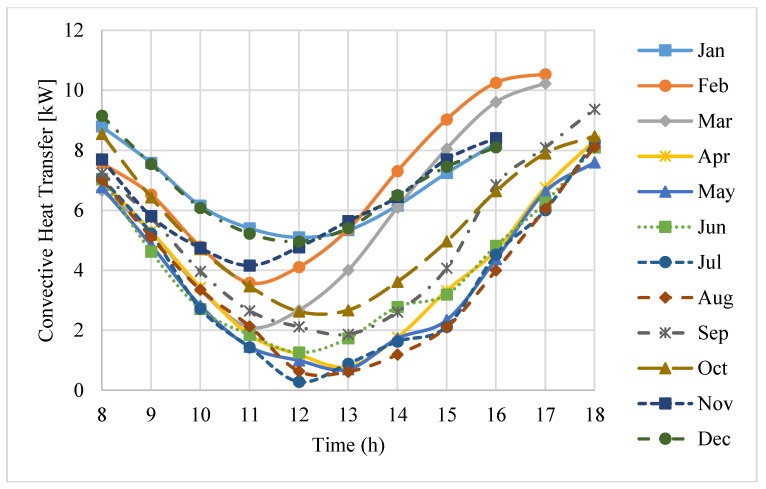
Convection losses during the daytime hours on an average day of each month.

Convection losses are affected by the orientation of the receiver aperture, the receiver cavity temperature, ambient temperature, and the wind speed. The total convection losses through the receiver cavity in daytime on an average day of each month in a year are shown in Figure 7. 

It can be seen that the convection loss is highly affected by the time of day. The minimum convection loss occurs at about midday. In addition, in the short period of time around midday, the convection loss changes only slightly with time. In contrast, when the time approaches early morning or late evening, the convection loss increases considerably. The main reason for this is that the orientation of the receiver has the main effect on the convection loss. At midday, the receiver aperture is facing downward, and its orientation is more vertical; whereas, during early morning or later evening, it is oriented more horizontally. Consequently, the receiver is subjected to a more stable convective situation at midday. Moreover, it can be seen that, during the interval of November to February, the convection loss is relatively higher. The main reason for this is that the convection loss is most affected by the time of the year due to changes in the solar incident angle. Consequently, changes in the orientation of the receiver aperture are unavoidable.

### 3.4. Radiation Loss through the Receiver

Radiative heat transfer contributes to thermal losses from the receiver through the aperture. This involves both radiation being emitted from the receiver aperture and radiation being reflected off the receiver cavity surfaces. Both forms of radiation loss are taken into account in the radiation model.

**Figure 8:**
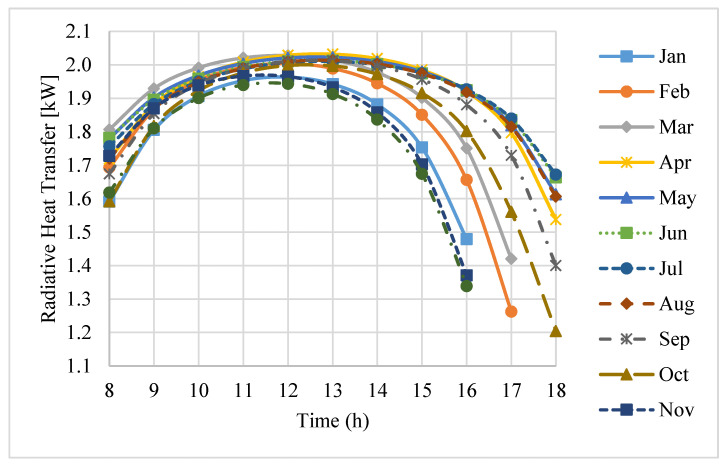
Radiation losses during the daytime hours on an average day of each month.

Figure 8 illustrates the total radiation loss during working hours on an average day of each month in a year. It can be seen that, in contrast to the convection loss, the radiation loss is the greatest at about midday, and it decreases in the early morning and later evening. Moreover, the radiation loss changes significantly during the day. In addition, unlike for convection loss, during the period from November to February, the radiation loss is relatively low.

### 3.5. Total Thermal Losses through the Receiver

Heat transfer mechanisms, including conduction, convection, and radiation, contribute to the total thermal energy losses from the receiver. The conduction loss has the smallest fraction among all of the thermal loss mechanisms. In early morning and late evening, the convection loss is dominant, which results in a considerable decrease in the thermal performance of the receiver while the convection loss decreases considerably during the midday period. In contrast to the trend of the convection loss, in the midday period, the radiation loss is high while it is lower in the early morning and later evening.

**Figure 9:**
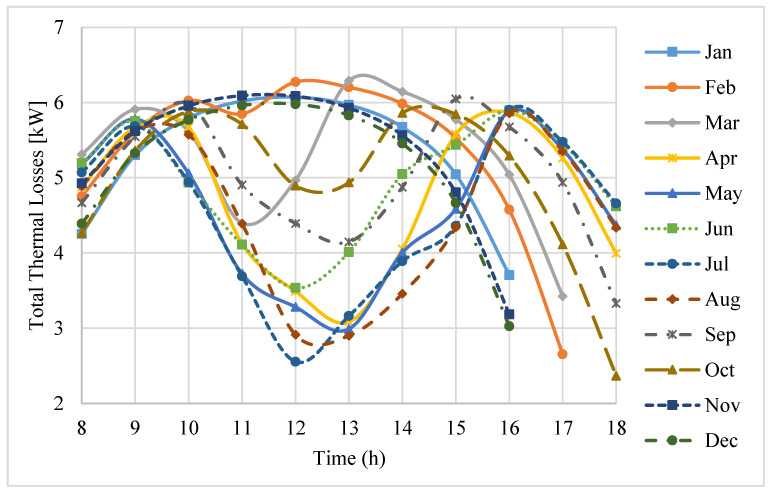
Total thermal losses during the daytime hours on an average day of each month.

Figure 9 illustrates the total thermal losses through the receiver in the working hours on an average day of each month in a year. It is found that, in early morning and late evening, the total heat losses are generally high, while, according to Figure 5, during this period of time, the flux intensities intercepted by the receiver are relatively low. Consequently, the receiver performance is minimal in early morning and late evening. In addition, between April and September, during midday, the receiver performance is considerably higher because the total thermal losses in these periods are comparatively low. On the other hand, in the other months (October to March), in the same daytime hours, the receiver performance is lower. This is the result from the comparatively higher total thermal losses during this period of time. It is found that, through the period of time between November to February, on one hand almost all of the power received by the receiver is unexploited due to the thermal losses. On the other hand, according to Figure 5, at the corresponding time, the flux intensity received by the receiver aperture is comparatively low. Consequently, the amount of thermal losses at the receiver aperture during this time results in the conclusion that the aperture diameter can be reduced by some millimeters to decrease the radiation and convective losses. Moreover, during the period of time in which the flux intensity received by the receiver aperture is comparatively high (May to August), the power received by the receiver can be increased by improving the optical quality of the dish.

### 3.6. Thermal Efficiency of the Receiver and the System Performance

**Figure 10:**
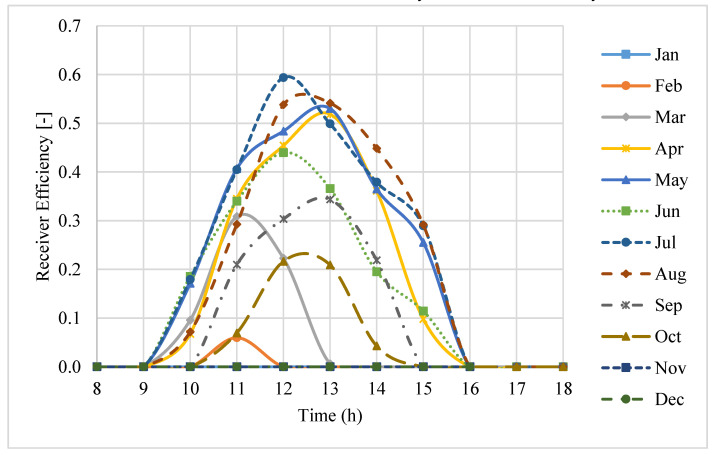
Thermal efficiency of the receiver during the daytime hours on an average day of each month.

Figure 10 shows the thermal efficiency of the receiver for the working hours on an average day of each month in a year. It can be seen that the receiver efficiency values decrease significantly in early morning and later evening. The maximum receiver efficiency was predicted to be about 60% at midday in July, while during some months of the year (November to February), the receiver efficiency is negligible. The main reason is that during this period of time, a major fraction of the thermal energy intercepted by the receiver is diminished by the thermal losses, while the incident flux intensity intercepted by the receiver aperture is relatively low.

**Figure 11:**
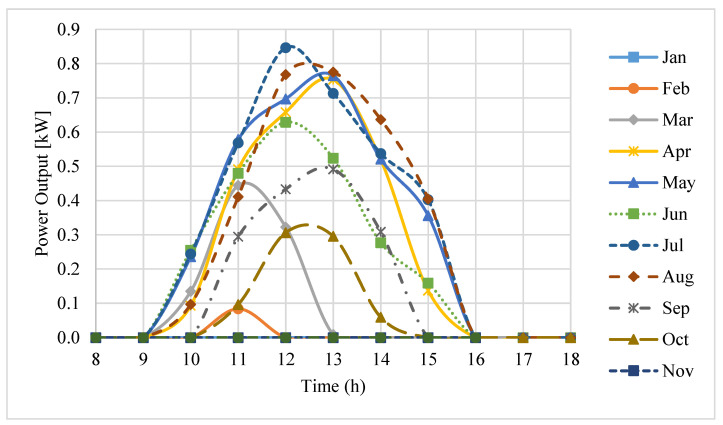
Power output of the Kerman pilot during the daytime hours on an average day of each month.

Finally, the predicted power outputs during the working hours of an average day on each month in a year are shown in Figure 11. A power output of 629 W was predicted by the model for midday in the middle of June. The Kerman pilot produced about 600 W power output at the same time, which shows that the predicted result and measured data were in close agreement. The power output also reached a maximum value of 850 W at midday in the middle of July.

## 4. Conclusions

The study developed a practical methodology which estimates the performance of the solar dish–Stirling system by calculating the performance of each part of the system. A practical example reflected important conclusions regarding the obtained results for thermal losses through the receiver, its thermal efficiency, and the power output. Thermal modeling and simulations for a standalone pilot parabolic solar dish–Stirling system composed of a 1-kW Stirling engine, a parabolic dish, and a receiver were conducted. The conduction, convection, and radiation heat losses through the receiver and their impact on its thermal efficiency for the selected design of the pilot system were examined. The pilot was set up in Kerman City, Iran. The input energy received by the receiver aperture, thermal losses through the receiver, thermal efficiency of the receiver, and the power output were predicted using an analytical model. The thermal losses resulting from the conduction, convection, and radiation heat transfer were calculated for the daytime hours on an average day of each month in a year. The results demonstrated that the convection loss was primary during early morning and later evening hours, which resulted in a significant decrease in the receiver performance during these periods of time. In contrast, the radiation loss played a more significant role in reducing the receiver performance during the midday period. The conduction loss also made the smallest contribution among all of the thermal losses. The receiver thermal efficiency values in the working hours on an average day of each month in a year were also calculated. Results showed that during early morning and later evening, the receiver efficiencies were minimal. It was found that during these periods, the convection losses were considerably higher, while the incident flux intensity values intercepted by the receiver aperture were comparatively lower. This resulted in poor receiver efficiency. For the given ambient conditions throughout the year, a maximum receiver efficiency of about 60% was estimated. The power output also reached a maximum of 850 W. 

It can be concluded that the approach presented in this study is useful to illustrate the influence of each of the thermal mechanisms contributing to thermal losses from the receiver, on its performance at different periods throughout a year. The methodology provided a comprehensive design procedure by taking into account thermal mechanisms, the efficiency of each element of the system, and the power output at different periods of time. As perspectives, the work presented a practical design approach that was able to make a whole-year simulation. The methodology can also be used to design more efficient and feasible systems.

## Nomenclature

A	area [m^2^]
C	concentration ratio [-]
d	diameter [m]
f	focal distance [m]
G	beam solar irradiance [W/m^2^]
Gr	Grashof number [-]
h	convective heat transfer coefficient [W/(m^2^ K)]
k	thermal conductivity [W/(m K)]
L	length [m]
Nu	Nusselt number [-]
Q	distance between the surface of the concentrator and the focal point [m]
$\dot{Q}$	heat transfer rate [W]
T	temperature [K]
v	wind speed [m/s]
**Greek symbols**	
α	absorptivity [-]
δ	thickness [m]
ε	emissivity [-]
η	efficiency [-]
θ	incident angle [deg]
σ	Stefan–Boltzmann constant [W/(m^2^ K^4^]
ψ_rim_	rim angle [deg]
**Subscripts**	
amb	ambient
ap	aperture
cav	receiver cavity
conc	concentrator
d	dish
eff	effective
insul	insulation
rec	receiver
